# Neoadjuvant therapy versus upfront surgery in resectable pancreatic cancer according to intention-to-treat and per-protocol analysis: A systematic review and meta-analysis

**DOI:** 10.1038/s41598-019-52167-9

**Published:** 2019-10-30

**Authors:** Yoon Suk Lee, Jong-Chan Lee, Se Yeol Yang, Jaihwan Kim, Jin-Hyeok Hwang

**Affiliations:** 1 Department of Internal Medicine, Inje University College of Medicine, Ilsan Paik Hospital, Goyang, Republic of Korea; 2 Department of Internal Medicine, Seoul National University College of Medicine, Seoul National University Bundang Hospital, Seongnam, Republic of Korea

**Keywords:** Surgical oncology, Gastrointestinal diseases

## Abstract

The effectiveness of neoadjuvant therapy (NAT) remains unclear in resectable pancreatic cancer (PC) as compared with upfront surgery (US). The aim of this study was to investigate the survival gain of NAT over US in resectable PC. PubMed and EMBASE were searched for studies comparing survival outcomes between NAT and US for resectable PC until June 2018. Overall survival (OS) was analyzed according to treatment strategy (NAT *versus* US) and analytic methods (intention-to-treat analysis (ITT) and per-protocol analysis (PP)). In 14 studies, 2,699 and 6,992 patients were treated with NAT and US, respectively. Although PP analysis showed the survival gain of NAT (HR 0.72, 95% CI 0.68–0.76), ITT analysis did not show the statistical significance (HR 0.96, 95% CI 0.82–1.12). However, NAT completed with subsequent surgery showed better survival over US completed with adjuvant therapy (HR 0.82, 95% CI 0.71–0.93). In conclusion, the supporting evidence for NAT in resectable PC was insufficient because the benefit was not demonstrated in ITT analysis. However, among the patients who completed both surgery and chemotherapy, NAT showed survival benefit over adjuvant therapy. Therefore, NAT could have a role of triaging the patients for surgery even in resectable PC.

## Introduction

Pancreatic cancer (PC) is one of the most lethal malignant tumors. The overall 5-year survival rate for all stages is approximately 8% in the US (National Cancer Institute. Surveillance, Epidemiology, and End Results (SEER) Program [http://seer.cancer.gov/]). More than 80% of patients are ineligible for curative surgery because they usually have distant metastasis or major vessel invasions at the time of diagnosis. If the pancreatic cancer is assessed to be “resectable,” curative resection is recommended as the primary treatment option. However, the overall recurrence rate is as high as 85% and most recurrences occur as systemic liver metastasis with a median disease-free survival of 6.7 months^1^, thus it leads to the necessity of early implementation of systemic therapy^2^. Early systemic therapy has been adopted to neoadjuvant therapy (NAT) in borderline resectable PC based on the findings of several randomized controlled trials (RCTs) demonstrating its survival benefit^3^. Nowadays, it is well accepted in the National Comprehensive Cancer Network guideline. Moreover, NAT strategy also has emerged as an attractive option even in resectable PC because of its potential benefits, including early treatment of occult micrometastasis and appropriate delivery of anticancer therapy that is not hampered by postoperative complications^4^. However, NAT may also be associated with the potential loss of eligibility for curative resection; presurgical attrition occurs in approximately 30% of patients with resectable PC, suggesting the possibility of selection bias in studies showing the benefits of NAT^4,5^. The real effectiveness of NAT in resectable PC remains unclear, with conflicting results on survival gain compared with upfront surgery (US)^6,7^. Therefore, this meta-analysis aimed to investigate whether the effectiveness of NAT is superior to that of US in patients with resectable PC. To minimize selection bias, we conducted subgroup analyses according to treatment strategy (NAT completed with surgical resection *versus* US completed with adjuvant therapy) and analytic method (intention-to-treat analysis (ITT) and per-protocol analysis (PP)).

## Methods

### Literature search

This systematic review was conducted according to the recommendations of the Preferred Reporting Items for Systematic Reviews and Meta-Analyses^8^. A comprehensive computerized database search of PubMed and EMBASE until the end of June 2018 was performed for all relevant studies comparing survival outcomes between NAT and US for patients with resectable PC published in any languages without the restriction of publication date. In addition, a manual search of the bibliographies of included trials and related reviews for additional references was conducted as well. The following keywords and their Medical Subject Heading terms were used for resectable PC: ((pancreatic OR pancreas) AND (cancer OR adenocarcinoma OR neoplasm OR tumor OR neoplasms)) AND (resectable OR resectability OR operable OR operability) AND (Neoadjuvant OR neo-adjuvant OR Preoperative OR pre-operative) AND survival (see Supplementary Table 1).

### Inclusion and exclusion criteria

All studies comparing survival outcomes between NAT and US in patients with resectable PC were deemed eligible for this meta-analysis. Because randomized controlled trials regarding this topic were rarely conducted, retrospective studies and nationwide population-based studies, such as the National Cancer Database (NCD) study, were also included if they reported the survival outcomes of NAT and US. The inclusion criteria were as follows: (1) the study enrolled patients assessed to have resectable PC; (2) the study enrolled a case group of patients treated with NAT and a comparator group of patients treated with US; (3) the outcomes were compared in terms of OS; and (4) sufficient information is available to estimate the hazard ratio (HR) and 95% confidence interval (CI). The eligible studies had to provide HR or crude data, and corresponding standard errors (SE), variance, CIs, or P value of the significance of the estimates. Otherwise, the studies should have to show the survival curves with the number in each group to estimate the HR^9,10^.

The exclusion criteria were as follows: (1) patients with borderline resectable PC were included for the survival outcome analysis; (2) patients with periampullary carcinoma arising from the ampulla of Vater or distal common bile duct were included for the survival outcome analysis; (3) single-arm design that did not have a control group; (4) case reports or only abstracts from conference meetings that were not published as original article; and (5) more than 1 report by the same author or working groups within the same study period.

### Data extraction

Three investigators (YS Lee, JC Lee, and SY Yang) independently reviewed the full manuscript of eligible studies and recorded information, including study design, resectability status of enrolled patients, inclusion and exclusion criteria, intervention type details (chemotherapy regimens, neoadjuvant chemotherapy, adjuvant chemotherapy, and radiation therapy or not), outcomes (overall survival and radical resectability), analysis type (ITT or PP), and timing of systemic therapy relative to the pancreatic resection (neoadjuvant or adjuvant). For studies with missing or ambiguous data, if possible, the authors contacted the first or corresponding author via telephone or email to collect the missing data.

### Risk of bias and quality assessments

This meta-analysis included RCTs and non-RCTs, and thus the Methodological Index for Non-Randomized Studies (MINORS) scale was used to assess the quality assessment of these studies. MINORS is a well-validated quality assessment tool for observational and non-randomized studies because prospective randomization could not be always possible or feasible, particulary in the surgical specialties^11^.

### Data synthesis and meta-analysis

The primary end point was OS, while the secondary end points were the proportion of resection rate, R0 resection rate, and lymph node metastasis after NAT. Whenever possible, the HR with 95% CI were obtained directly from each study or were calculated from the reported data using the method proposed by Parmar *et al*.^9^. When HRs were not reported, it was estimated from the Kaplan-Meier survival curves using the method described by Tierney *et al*.^10^. The Chi-square- based Q-test and I^2^ statistics test were used to assess the heterogeneity of studies. Statistically significant heterogeneity was considered if P was <0.1 or the I^2^ statistic was >50%^12^. Sources of heterogeneity were investigated via sensitivity analysis. Furthermore, subgroup analysis was conducted according to the analysis approach: ITT or PP. A funnel plot was constructed by plotting the inverse of the SE against the log HR to qualitatively assess publication bias. All analyses were performed with RevMan software (version 5.3, Nordic Cochrane Center, Copenhagen, Denmark).

## Results

**Figure 1:**
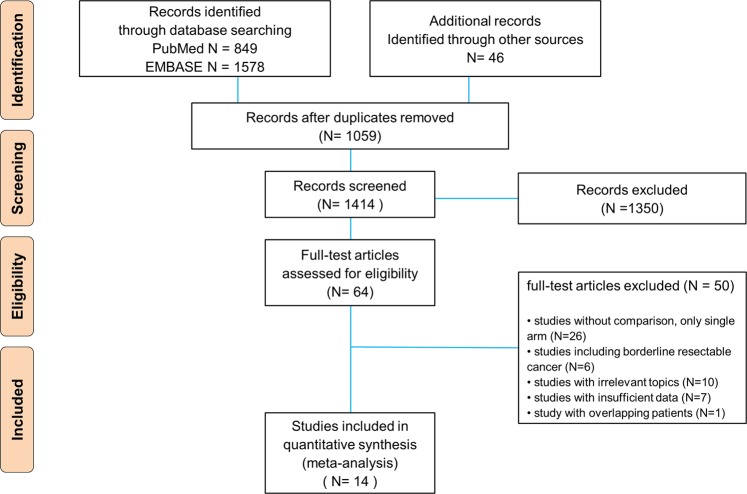
Flow diagram for identification of selected studies in the meta-analysis.

A total of 2,473 records were initially retrieved from computerized database search and manual checking. After removal of duplicate records, 1,414 records were considered for reviewing the title and abstract. Of these, 64 records were selected as seemingly relevant publications. After assessment of full text for eligibility, 50 records were further excluded because they were single-arm observation studies (n = 26), included borderline resectable pancreatic cancers in the survival analysis (n = 6), irrelevant topics (n = 10), had insufficient information (n = 7), and had overlapping patient groups (n = 1). Finally, 14 studies were included in the pooled analysis (Fig. 1).

### Description of included studies

The 14 studies included 2,699 patients treated with NAT and 6,992 treated with US^13–26^. Four studies were conducted in the United States, 5 in Europe, and 5 in Asia. Only one of the studies was a prospective randomized trial, and it was terminated early due to a low accrual rate^22^. The other 13 studies were retrospective studies, including 2 population-based studies (NCDB and the Los Angeles Country Cancer Surveillance Program database). The main characteristics of the studies including their quality (MINORS) score and the anticancer therapy regimens are summarized (see Supplementary Table 2).

**Figure 2:**
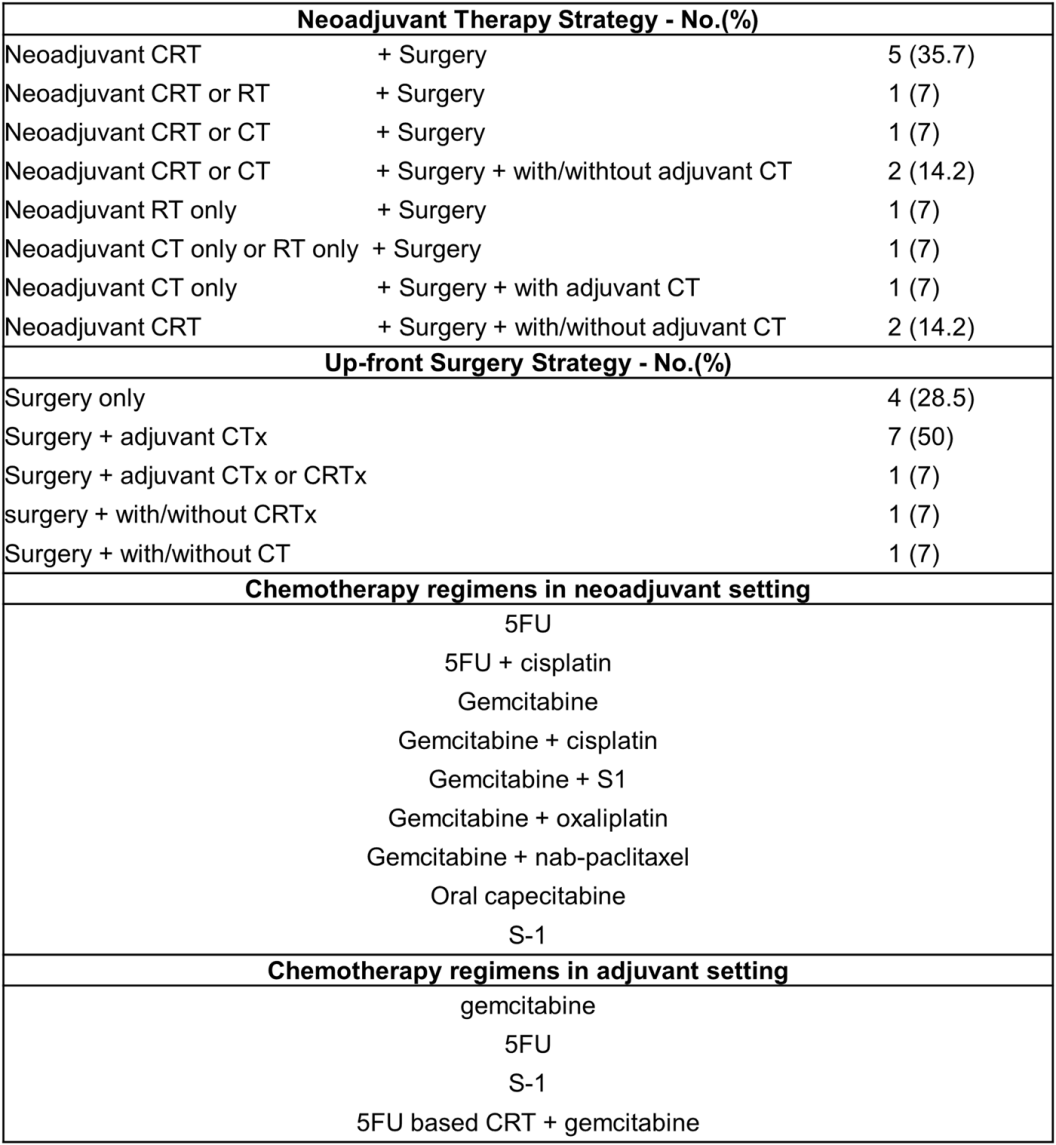
Anticancer therapy strategies and chemotherapy regimens in NAT and US strategy, respectively in the studies of this meta-analysis.

The most common NAT strategy was neoadjuvant chemoradiotherapy (CRT) followed by surgical resection. Although various chemotherapy regimens were used, gemcitabine- and 5-FU-based regimens were the mainstay of the regimens, which was administered as a monotherapy or combination therapy with other cytotoxic agents (cisplatin or S-1) (Fig. 2). Only one study by Ielpo *et al*. in 2017 used neoadjuvant chemotherapy with gemcitabine and nab-paclitaxel as NAT^26^. Neoadjuvant radiotherapy was used as NAT by Ishikawa *et al*. in 1994^13^.

### Survival difference between NAT and US

**Figure 3:**
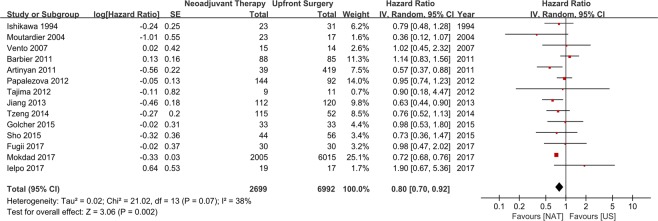
Meta-analyses of 14 studies on overall survival of NAT versus US using random effects model. NAT strategy had a better OS compared with US strategy in resectable pancreatic cancer (HR 0.80, 95% CI 0.70–0.92, *P* = 0.002). although the heterogeneity between studies is identified (Chi^2^ = 21.02, *P* = 0.070, I^2^ = 38%).

**Figure 4:**
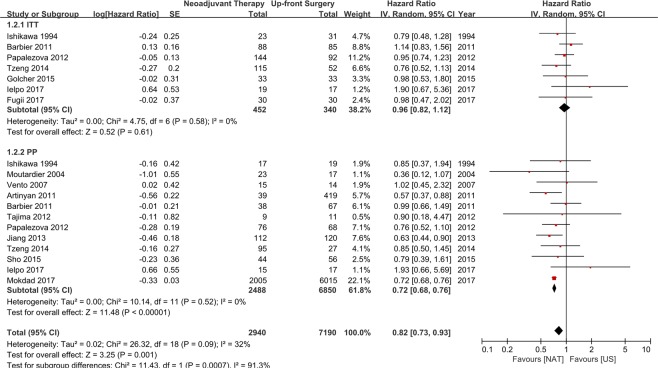
Subgroup analysis based on the analytic method of survival data (ITT or PP analysis). The subgroup analysis with PP analysis showed the survival gain of NAT (HR 0.72, 95% CI 0.68–0.76, *P* < 0.001). However, the studies with ITT analysis did not show the survival gain of NAT (HR 0.96, 95% CI 0.82–1.12, *P* = 0.610).

A meta-analysis of the 14 studies was conducted using a random effects model, and the results showed that NAT yields better OS than US in resectable PC (HR 0.80, 95% CI 0.70–0.92, *P* = 0.002). However, a significant heterogeneity was found between the enrolled studies (Chi^2^ = 21.02, *P* = 0.070, I^2^ = 38%) (Fig. 3). Therefore, subgroup analysis was conducted to evaluate the effects of analytic method, which were categorized into ITT and PP studies. ITT was defined as the inclusion of patients who received NAT but did not undergo surgical resection in the survival analysis, while PP was defined as the inclusion only of patients who underwent surgical resection after NAT in the survival analysis. Seven studies with ITT analysis and 12 studies with PP analysis were found, and 5 studies reported the survival outcomes of both ITT and PP analysis. The subgroup with PP analysis (12 studies; 2,488 patients in the NAT group, 6,850 patients in the US group) showed the survival gain of NAT (HR 0.72, 95% CI 0.68–0.76, *P* < 0.001). However, the studies with ITT analysis (7 studies; 452 patients in the NAT group, 340 patients in the US group) did not show the survival gain of NAT (HR 0.96, 95% CI 0.82–1.12, *P* = 0.610). Furthermore, there was no statistically significant heterogeneity within the studies in each subgroup (ITT subgroup: *P = *0.580, I^2^ = 0%; PP subgroup: *P* = 0.520, I^2^ = 0%) (Fig. 4).

Nonetheless, considering that the population-based study from Mokdad *et*. *al*. might play a role of skewing the data due to its large sample size, the pooled HR was reassessed after excluding the study and this sensitivity analysis still showed the favorable effect of NAT. In addition, further sensitivity analyses were performed to evaluate the influence of each enrolled study (see Supplementary Fig. S1).

### Timing of systemic therapy relative to pancreatic resection (neoadjuvant or adjuvant)

**Figure 5:**
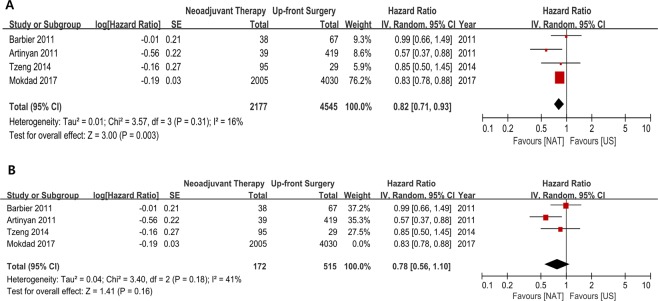
(**A**) Subgroup analysis regarding the delivery sequence showed that the neoadjuvant delivery of anticancer therapy had survival benefit over the adjuvant delivery among the patients underwent surgical resection (HR 0.82, 95% CI 0.71–0.93, *P* = 0.003) and the heterogeneity between studies was not significant (Chi^2^ = 3.57, *P* = 0.310, I^2^ = 16%). (**B**) Sensitivity analysis after excluding the study of Mokdad *et al*. showed that the trend of survival benefit of NAT still maintained.

Only 4 studies evaluated the effect of timing between NAT and adjuvant therapy for the patients who underwent surgical resection. A total of 2,177 and 4,545 patients were treated with NAT followed by resection and with US followed by adjuvant chemotherapy, respectively. The pooled meta-analysis showed that among those who underwent resection, NAT had superior survival benefit over US (HR 0.82, 95% CI 0.71–0.93, *P* = 0.003) and there was no significant heterogeneity between the studies (Chi^2^ = 3.57, *P* = 0.310, I^2^ = 16%) (Fig. 5A). Furthermore, considering the study of Mokdad *et al*. might be the main drive of skewing the data, the pooled HR was reassessed after excluding the study. This sensitivity analysis still showed that the trend of survival benefit of NAT was maintained although statistical power turned out to be insignificant (HR 0.78, 95% CI 0.56–1.10, *P = *0.160) (Fig. 5B).

### Resection rate and margin positivity

The secondary outcomes analyzed were the resection rate and resection margin status. A total of 8 studies were included in the analysis for the resection rate. The NAT and the US group comprised 501 and 560 patients, respectively. The resection rate was significantly lower in the NAT group than that in the US group (OR 0.46, 95% CI 0.25–0.85) in the ITT analysis, and there was a significant heterogeneity between the studies (Chi^2^ = 18.15, *P* = 0.010, I^2^ = 61%) (see Supplementary Fig. S2). The presurgical attrition rate was significantly higher in the NAT group than that in the US group (36.3% in the NAT group *versus* 17.3% in the US group).

A total of 8 studies were included in the analysis for resection margin status. The NAT and the US groups comprised 2,408 and 6,652 patients, respectively. The rate of R0 resection after surgery was significantly higher in the NAT group than that in the US group (OR 1.53, 95% CI 1.35–1.73) in the PP analysis, and there was no statistically significant heterogeneity between the included studies (Chi^2^ = 10.82, *P* = 0.230, I^2^ = 25%) (see Supplementary Fig. S3).

### Lymph node metastasis

A total of 11 studies were included in the analysis of lymph node metastasis. The NAT and the US group comprised 2,380 and 7,032 patients, respectively. The rate of LN metastasis was significantly lower in the NAT group than that in US group (OR 0.37, 95% CI 0.26–0.52, *P* < 0.001) in the PP analysis, although there was significant heterogeneity between the studies (Chi^2^ = 21.04, *P* = 0.02, I^2^ = 52%) (see Supplementary Fig. S4A). Therefore, for the sensitivity analysis, the results from the 2 studies by Ishikawa *et al*. and Tajima *et al*. were eliminated because these studies used only radiotherapy or chemotherapy as NAT, whereas the other studies used concurrent CRT. This sensitivity analysis showed that the favorable effect of NAT was still preserved (OR 0.32, 95% CI 0.24–0.43, *P* < 0.001), and the heterogeneity between studies statistically turned out to be insignificant (Chi^2^ = 11.78, *P* = 0.160, I^2^ = 32%) (see Supplementary Fig. S4B).

### Publication bias

There was no possible funnel asymmetry in the analyses of survival difference and lymph node status, while slight asymmetry was found for the analyses of resection failure and R0 resection with possible lack of negative effect studies (see Supplementary Fig. S5).

## Discussion

This pooled meta-analysis showed that the risk for overall mortality in patients with resectable PC was lower in NAT than that in US (HR 0.80, 95% CI 0.70–0.92, *P* < 0.01). Moreover, R0 resection was achieved more frequently (83.7% in NAT *versus* 76.8% in US), and lymph node metastasis occurred less frequently in NAT than that in US (45.0% in NAT *versus* 69.3% in US). However, the survival benefit of NAT turned out to be insignificant in the ITT analysis, and this might be due to the influence of the higher presurgical attrition rate in NAT than that in US (36.3% *versus* 17.3%). Nevertheless, among the patients who completed both pancreatic resection and chemotherapy, NAT appears to be more effective because it still showed a survival benefit over adjuvant therapy (HR 0.82, 95% CI 0.71–0.93, *P* < 0.01).

In 2016, a population-based retrospective study from the NCD demonstrated that NAT followed by resection was superior than US in resected pancreatic head cancer, thereby supporting the use of NAT for resectable PC^25^. The beneficial effect of NAT was explained by the fact that the delivery of anticancer therapy was not hampered by insufficient recovery or postoperative complications and early systemic treatment of occult micrometastasis, thereby improving pathologic outcomes after resection^27,28^. Similar favorable outcomes were also found in our meta-analysis. However, concerns about selection bias have been raised because of the high number of patients who did not undergo curative resection after NAT^29^. Therefore, to minimize selection bias, we further categorized the enrolled studies into two groups based on the analytic method of survival data (ITT or PP analysis). Subsequent analysis showed that the beneficial effect of NAT for OS was only in the PP analysis, but not in ITT analysis. This diminution may be influenced by the higher presurgical attrition rate after NAT than those in US. High presurgical attrition rates after NAT have also been reported in previous studies, including in high-volume tertiary medical centers^30,31^.

In this clinical context, another PP analysis (NAT followed by completion of pancreatic resection [2,177 patients] vs UP followed by completion of adjuvant therapy [4,545 patients]) was evaluated for direct comparison of the delivery timing of chemotherapy, and it showed that NAT still had survival benefit over adjuvant therapy (HR 0.82, 95% CI 0.71–0.93, *P* < 0.01), and there was no significant heterogeneity between studies (Chi^2^ = 3.57, *P* = 0.31, I^2^ = 16%).

Collectively, the results of our meta-analysis reveal that NAT strategy might help discriminate patients with aggressive tumor biology who will not benefit from direct resection. If the planned NAT with subsequent resection is successfully implemented, the survival outcome would be more favorable than that of US with adjuvant chemotherapy. Therefore, future researche is needed to develop the selection criteria for discriminating between patients who will gain a survival benefit with NAT or US. There are several ongoing prospective trials enrolling patients with resectable PC for NAT that use these combined regimens, such as gemcitabine and nab-paclitaxel or 5-FU, folinic acid, irinotecan, and oxaliplatin (FOLFIRINOX)^32,33^. The results of these trials are expected to clarify the effect of NAT in resectable PC.

There were several limitations in this pooled meta-analysis. First, majority of the enrolled studies were retrospective in study design. Given that patients may become ineligible for radical resection, no well-designed RCT has been conducted to date. Second, the NAT regimens in the enrolled studies were diverse, and some were not up-to-date. Furthermore, although FOLFIRINOX or gemcitabine/abraxane has been established as the most effective regimen for advanced PC with expanding indications into borderline resectable PC or adjuvant chemotherapy^33,34^, only one study using a combined chemotherapy regimen (gemcitabine/abraxane) was included in our meta-analysis.

However, the strength of our meta-analysis is that the possibility of selection bias was controlled by stratifying the enrolled studies into two groups according to the method of survival analysis (ITT or PP analysis) and the timing of systemic therapy relative to the pancreatic resection (neoadjuvant or adjuvant). Furthermore, unlike previous meta-analyses^6,7,35^, our study included only resectable PC.

Recently, Bradley *et al*. conducted a systemic review and meta-analysis of the effectiveness of NAT vs. US for resectable PC^36^. However, our and their studies differed in several aspects. First, we discriminated the analyses of ITT and PP, whereas Bradley *et al*. conducted a combined analysis. Accordingly, we identified that the survival benefit of NAT was insignificant in the ITT analysis but significant in the PP analysis, indicating that NAT provides a survival benefit only to patients who completed both scheduled surgery and chemotherapy. Second, the qualitative and quantitative syntheses performed by Bradley *et al*. may have involved considerable data overlap because two included studies by Mokdad *et al*.^25^ and de Geus *et al*.^37^ used the same NCD database and data collected during overlapping periods from 2006 to 2012. Furthermore, two other studies by Roland *et al*.^27^ and Tzeng *et al*.^21^ used the same MD Anderson Cancer Center database and data collected during overlapping periods from 2002 to 2007. In contrast, we eliminated the possibility of overlapping data from our meta-analysis by using only the abstracted NCD data used previously by Mokdad *et al*.^25^ and the MD Anderson Cancer Center data used previously by Tzeng *et al*.^21^ Third, we performed a more extensive literature search and included seven additional articles^13,14,16,17,19,20,23^. Finally, with respect to the technical aspects of a meta-analysis, Bradley *et al*. conducted a Bayesian network meta-analysis (BNMA), in which two different interventions were compared indirectly using another comparator^38^. Therefore, although Bradley and colleagues produced a high-quality BNMA, the results of their meta-analysis should be interpreted cautiously.

In conclusion, our study did not show sufficient evidence for survival benefit of NAT in resectable PC when compared with US, despite the favorable outcomes with respect to R0 resection rate, and the number of lymph nodes involved. However, NAT completed with subsequent resection showed significantly better survival benefit than US completed with adjuvant treatment. Collectively, our findings support that NAT could help triage the patients for surgery even in resectable PC.

## Supplementary information

Supplementary Information
