# Supplementary material for: Neoadjuvant therapy versus upfront surgery in resectable pancreatic cancer according to intention-to-treat and per-protocol analysis: A systematic review and meta-analysis
Source: Sci Rep. 2019 Oct 30;9:15662. doi: 10.1038/s41598-019-52167-9 (PMC6821820; doi:10.1038/s41598-019-52167-9)

Neoadjuvant therapy versus upfront surgery in resectable pancreatic cancer according to intention-to-treat and per-protocol analysis: A systematic review and meta-analysis

Yoon Suk Lee, MD[1], Jong-Chan Lee, MD[2], Se Yeol Yang, MD[2], Jaihwan Kim, MD[2], Jin-Hyeok Hwang, MD, PhD[2*]

[1]Department of Internal Medicine, Inje University College of Medicine, Ilsan Paik Hospital, Goyang, Republic of Korea, [2]Department of Internal Medicine, Seoul National University College of Medicine, Seoul National University Bundang Hospital, Seongnam, Republic of Korea

Table S1. Electronic search criteria for systematic review of literature

Database	Search criteria	Date
PubMed	((("pancreas"[MeSH Terms] OR "pancreas"[All Fields] OR "pancreatic"[All Fields]) OR ("pancreas"[MeSH Terms] OR "pancreas"[All Fields])) AND (("neoplasms"[MeSH Terms] OR "neoplasms"[All Fields] OR "cancer"[All Fields]) OR ("adenocarcinoma"[MeSH Terms] OR "adenocarcinoma"[All Fields]) OR ("neoplasms"[MeSH Terms] OR "neoplasms"[All Fields] OR "neoplasm"[All Fields]) OR ("tumour"[All Fields] OR "neoplasms"[MeSH Terms] OR "neoplasms"[All Fields] OR "tumor"[All Fields]) OR ("neoplasms"[MeSH Terms] OR "neoplasms"[All Fields]))) AND (resectable[All Fields] OR resectability[All Fields] OR operable[All Fields] OR operability[All Fields]) AND (("neoadjuvant therapy"[MeSH Terms] OR ("neoadjuvant"[All Fields] AND "therapy"[All Fields]) OR "neoadjuvant therapy"[All Fields] OR "neoadjuvant"[All Fields]) OR neo-adjuvant[All Fields] OR Preoperative[All Fields] OR pre-operative[All Fields]) AND ("mortality"[Subheading] OR "mortality"[All Fields] OR "survival"[All Fields] OR "survival"[MeSH Terms]	30 June 2018
EMBASE	(pancreatic OR 'pancreas'/exp OR pancreas) AND ('cancer'/exp OR cancer OR 'adenocarcinoma'/exp OR adenocarcinoma OR 'neoplasm'/exp OR neoplasm OR 'tumor'/exp OR tumor OR 'neoplasms'/exp OR neoplasms) AND (resectable OR resectability OR operable OR operability) AND (neoadjuvant OR 'neo adjuvant' OR preoperative OR 'pre operative')	30 June 2018

Table S2. Main characteristics of the 14 studies in the meta-analysis

	Study	Year	Country	Design	Institution	Pancreatic caner location	Study Period	Arm	NAT regimen		Adjuvant therapy	Time Interval between surgery and NAT or adjuvant therapy
									Radiation dose, fractionation	Chemotherapy		
1	Ishikawa, et al[13]	1994	Japan	Retrospective, case-control study	The Center for Adult Disease, Osaka	Pancreas head cancer only	1985-1989	Neoadjuvant RT only with subsequent surgery	50 Gy, 25 fractions for 5 weeks of 10 MeV of x-ray	None	None	NA
								Upfront surgery only	None	None	None	
2	Moutardier, et al[14]	2004	France	Retrospective, case-control study	The Universite de la Mediterranee, Marseille	Pancreas head cancer only	1997-2002	Neoadjuvant CRT with subsequent surgery	45 Gy, 1.8 Gy per fraction (25 fraction) 5 days a week (5 weeks of 15 MeV photons)	5-FU 650 mg/m^2 on day 1 to 5 and days 21 to 25 + Cisplatin 80 mg/m^2 on days 2 and 22	None	Median: 43 days (range 10 to 90 days) from the last of NAT to surgery
								Upfront surgery only	None	None	None	
3	Vento, et al[15]	2007	Finland	Retrospective, case-control study	The Helsinki University Central Hospital, Helsinki	Pancreas head cancer only	1999-2002	Neoadjuvant CRT with subsequent surgery	50.4 Gy, 28 fraction of 1.8 Gy per fraction, 5 days a week	Gemcitabine 50 mg/m^2	None	NA
								Upfront surgery only	None	None	None	

4	Barbier, et al[16]	2011	France	Retrospective, case-control study, multicenter	La Conception Hospital, Marseille, France.	Pancreas head cancer only	1997-2006	Neoadjuvant CRT with subsequent surgery	45 Gy, 1.8 Gy per fraction (25 fraction) 5 days a week	5-FU 650 mg/m^2 on day 1 to 5 and days 21 to 25 + Cisplatin 80 mg/m^2 on days 2 and 22	None	Median: 4 months (range 2-7 months) from diagnosis to surgery
					Paoli-Calmettes Institue, Mediterranean University, Marseille			Upfront surgery with adjuvant CT	None	None	5-FU-leucovorine 400mg/m^2 bolus & 600mg/m^2 22hr infusion on day 1 & 2, every 2 weeks or gemcitabine1000mg/m^2	NA
5	Artinyan, et al[17]	2011	United States	Retrospective, case-control study	LA County Cancer Surveillance Program Database	Pancreas head & body/tail	1987-2006	Neoadjuvant RT or CRT with subsequent surgery	Not mentioned in detail regimen	Not mentioned in detail regimen		NA
								Upfront surgery with adjuvant CT or CRT	None	None	not mentioned in detail regimen	NA
6	Papalezova, et al[18]	2012	United States	Retrospective, case-control study	Duke University Medical Center	Pancreas head cancer only	1999-2007	Neoadjuvant CRT + surgery with/without adjuvant CT	45 Gy, 1.8 Gy er fraction, 5 days for 5 weeks	5-FU based (oral capecitabine or infusion 5-FU)	Gemcitabine 1000mg/m^2	Median: 3 months (range 0-7months) between diagnosis and resection

								Upfront surgery with/without adjuvant CRT and followed by CT	None	None	5-FU based CRT followed by gemcitabine	NA
7	Tajima, et al[19]	2012	Japan	Retrospective, case-control study	Kanazawa University Hospital	Pancreas head & body/tail	2006-2009	Neoadjuvant CT only + surgery with adjuvant CT	None	S1 30mg/m^2 at day 1 to 15 with gemcitabine 800mg/m^2 at day 8 and day 15 per 2 weeks	gemcitabine 1000mg/m2	NA
								Upfront surgery with adjuvant CT	None	None	Gemcitabine 1000mg/m2	NA
8	Jiang, et al[20]	2013	China	Retrospective, case-control	The Second People's Hospital of Neijiang, Luzhou Medical College, Neijiang, Sichuan	Pancreas head & body/tail	2004-2010	Neoadjuvant CT only or RT only + surgery	46-50 Gy, 23 fractions	5-FU 600mg/m^2 at D1, D8, D16 or gemcitabine 1000mg/m^2 at D1, D8, D15 or gemcitabine with oxaliplatin	None	NA
								Upfront surgery only	None	None	None	
9	Tzeng, et al[21]	2014	United States	Retrospective, case-control study, prospectively maintained pancreatic translational database	The University of Texas MD Anderson Cancer Center	Pancreas head cancer only	2002-2007	Neoadjuvant CRT + surgery	rapid fractionation external-beam radiation (30 Gy) with gemcitabine or [CRT alone case; 30-50 Gy with radiosensitizing 5-FU or gemcitabine]	CT with CRT (gemcitabine and cisplatin CT + rapid fractionation external-beam radiation (30 Gy) with gemcitabine		NA

								Upfront surgery with adjuvant CT	None	None	Not mentioned in detail regimen	NA
10	Golcher, et al[22]	2015	Germany	prospective, randomized trial (early termination due to low accrual rate)	University Hospital Erlangen, Freiburg, eight hospitals from Germany and Switzerland	Pancreas head cancer only	2003-2009	Neoadjuvant CRT + surgery with/without adjuvant CT	55.8 Gy or 50.4 Gy	Gemcitabine 300 mg/m^2 and cisplatin 30mg/m^2 D1,D8,D22	Gemcitabine 1000mg/m^2 (from 2005)	NA
								Upfront surgery with adjuvant CT	None	None	Gemcitabine 1000mg/m^2 (from 2005)	NA
11	Sho, et al[23]	2015	Japan	Retrospective, case-control study	Nara Medical University Hospital	Pancreas head cancer only	2006-2013	Neoadjuvant CRT or CT + surgery with/without adjuvant CT	50-54Gy, 25 fractions, 10 MeV	Gemcitabine 1000mg/m^2 (for CRT); Gemcitabine 1000mg/m^2 D1,8 and S-1 40 mg/m^2 for 14 consecutive days	High dose 5-FU hepatic artery infusion and Gemcitabine	NA
								Upfront surgery with/without adjuvant CT	None	None	High dose 5-FU hepatic artery infusion and gemcitabine	NA
12	Fugii, et al[24]	2017	Japan	Retrospective, prospectively maintained database, propensity	Two reginal high-volume centers (Nagoya University	Pancreas head cancer only	2001-2013	Neoadjuvant CRT (50.4 Gy + oral S1) + surgery	50.4 Gy, 28 fraction	Oral S-1 80mg/m^2 D1~D14, D22~35		NA

				matching	Graduate School of Medicine, Kansai Medical University)			Upfront surgery with adjuvant CT	None	None	Gemcitabine 1000mg/m^2 or S-1 80mg/m^2 D1-28 (2wks rest)	NA
13	Mokdad, et al[25]	2017	United States	Retrospective, National Cancer database (NCDB) analysis, propensity matching	NCD database analysis (University of Texas Southwestern Medical Center)	Pancreas head cancer only	2006-2012	Neoadjuvant CRT or CT + surgery	Not mentioned in detail regimen	Not mentioned in detail regimen	None	NA
								Upfront surgery with or without adjuvant CT	None	None	Not mentioned in detail regimen	NA
14	Ielpo, et al[26]	2017	Spain	Retrospective, prospectively maintained database	Sanchinarro University Hospital, Madrid	Pancreas head & body/tail	2007-2016	Neoadjuvant CT + RT since 2013 + surgery (2011 Aug ~ 2016 March) with adjuvant Gem	52 Gy, 5 days per week (since 2013)	gemcitabine 1000mg/m^2+Nab-paclitaxel 125 mg/m^2 (D1,D8,D15 for 28 days)	Gemcitabine 1000mg/m2	NA
								Upfront surgery only (2007-2011) with adjuvant CT (2012-2016)	None	None	Gemcitabine 1000mg/m^2	NA

CT: chemotherapy, CRT: chemoradiotherapy, Gem: gemcitabine, D; day, NA: not available

Table S2 (continued).

	Study	Year	Analytic method	Arm	No. pts of each analysis		Surgical resection	R0 resection	Node positive	MINORS score
					ITT	PP				
1	Ishikawa, et al[13]	1994	ITT & PP	Neoadjuvant RT only with subsequent surgery	23	17	17/23(73.9%)	Not mentioned	5/17 (29.4%)	16
				Upfront surgery only	31	19	19/31(61.2%)		4/18 (22.2%)	
2	Moutardier, et al[14]	2004	ITT & PP	Neoadjuvant CRT with subsequent surgery	39	23	23/39(58.9%)	21/23 (91.3%)	3/23 (13%)	17
				Upfront surgery only	17	17	17/17 (100%)	11/17 (64.7%)	11/17 (64.7%)	
3	Vento, et al[15]	2007	PP	Neoadjuvant CRT with subsequent surgery		15	enrolled after resection	Not mentioned	7/15(46.6%)	18
				Upfront surgery only		14			5/14(35.7%)	

4	Barbier, et al[16]	2011	ITT & PP	Neoadjuvant CRT with subsequent surgery	88	38	38/88(43.1%)	35/38 (92.1%)	11/38 (29%)	18
				Upfront surgery with adjuvant CT	85	67	67/85(78.8%)	45/67 (67.1%)	42/66 (64%)	
5	Artinyan, et al[17]	2011	PP	Neoadjuvant RT or CRT with subsequent surgery		39	enrolled after resection	Not mentioned	16/39 (44.4%)	18
				Upfront surgery with adjuvant CT or CRT		419			255/419 (65.9%)	
6	Papalezova, et al[18]	2012	ITT & PP	Neoadjuvant CRT + surgery with/without adjuvant CT	144	76	76/144(52.8%)	59/76(78%)	19/76 (25%)	19
				Upfront surgery with/without adjuvant CRT and followed by CT	92	68	68/92(73.9%)	54/68(79%)	42/68 (62%)	
7	Tajima, et al[19]	2012	PP	Neoadjuvant CT only + surgery with adjuvant CT		9	enrolled after resection	11/13 (84.6%)	10/13 (76.9%)	17

				Upfront surgery with adjuvant CT		11		18/21 (85.7%)	12/21 (57.1%)	
8	Jiang, et al[20]	2013	PP	Neoadjuvant CT only or RT only + surgery		112	enrolled after resection	95/112 (84.8%)	not mentioned	17
				Upfront surgery only		120		96/120(80.0%)		
9	Tzeng, et al[21]	2014	ITT & PP	Neoadjuvant CRT + surgery	115	95	95/115(82.6%)	85/95(89.4%)	49/95 (51.6%)	20
				Upfront surgery with adjuvant CT	52	29	48/52(92.3%)	39/48 (81.2%)	39/48 (81.2)	
10	Golcher, et al[22]	2015	ITT	Neoadjuvant CRT + surgery with/without adjuvant CT	33	19	19/33(57.5%)	17/19(89.4%)	6/19 (31.6%)	22
				Upfront surgery with adjuvant CT	33	23	23/33(69.6%)	16/23(69.5%)	13/23 (56.5%)	

11	Sho, et al[23]	2015	PP	Neoadjuvant CRT (54 Gy + Gem) or CT (Gem + S1) + surgery with/without adjuvant CT		44	enrolled after resection	43/44 (98%)	not mentioned	17
				Upfront surgery with/without adjuvant CT		56		50/56 (89%)		
12	Fugii, et al[24]	2017	ITT	Neoadjuvant CRT + surgery	30 after propensity matching of 40		36/40(90.0%)	34/40(86%)	14/40 (39%)	20
				Upfront surgery with adjuvant CT	30 after propensity matching of 233		204/233(87.5%)	163/204(70%)	145/233 (71%)	
13	Mokdad, et al[25]	2017	PP	Neoadjuvant CRT or CT + surgery		2005	enrolled after resection	1670/2005(83.2%)	932/2005 (48%)	18
				Upfront surgery with or without adjuvant CT		6015		4688/6105 (76.7%)	4306/6105 (73%)	

14	Ielpo, et al[26]	2017	ITT & PP	Neoadjuvant CT + RT since 2013 + surgery (2011 Aug ~ 2016 March) with adjuvant CT	19	15	15/19 (79%)	not mentioned	not mentioned	18
				Upfront surgery only (2007 ~ 2011 Oct) with adjuvant CT	17	17	17/17 (100%)			

CT: chemotherapy, CRT: chemoradiotherapy, Gem: gemcitabine, D: day, NA: not available

Figure S1. Sensitivity analysis still showed the favorable effect of NAT. The pooled HR was reassessed after excluding the named study in each line.

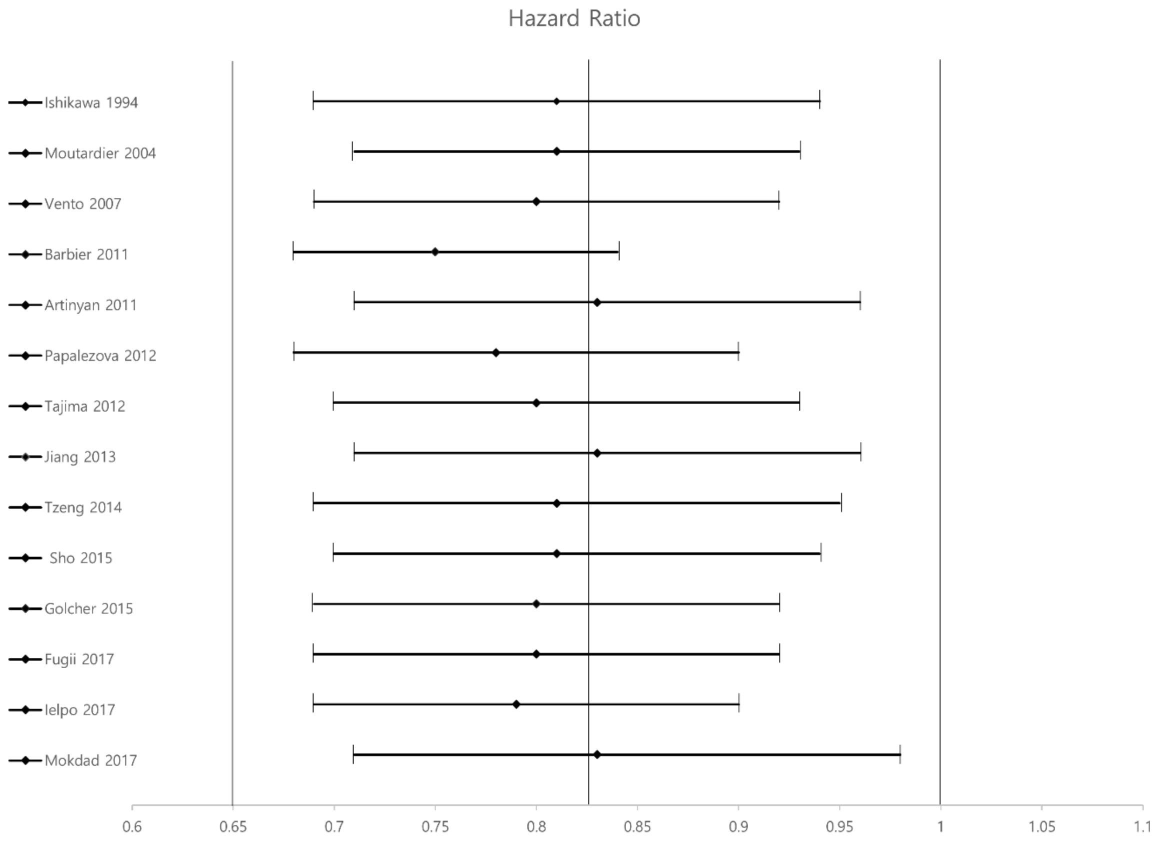

Figure S2. The rate of undergoing surgical resection was significantly lower in NAT group compared with US group (OR 0.46, 95% CI 0.25-0.85), although there was significant heterogeneity between studies ($Chi^2 = 18.15$, $P = 0.010$, $I^2 = 61\%$).

	Neoadjuvant Therapy		Up-front Surgery			Odds Ratio	
Study or Subgroup	Events	Total	Events	Total	Weight	M-H, Random, 95% CI	Year
Ishikawa 1994	17	23	19	31	12.8%	1.79 [0.55, 5.81]	1994
Moutardier 2004	23	39	17	17	3.9%	0.04 [0.00, 0.73]	2004
Barbier 2011	38	88	67	85	18.6%	0.20 [0.10, 0.40]	2011
Papalezova 2012	76	144	68	92	19.8%	0.39 [0.22, 0.70]	2012
Tzeng 2014	95	115	48	52	13.3%	0.40 [0.13, 1.22]	2014
Golcher 2015	19	33	23	33	14.5%	0.59 [0.21, 1.63]	2015
Ielpo 2017	15	19	17	17	3.6%	0.10 [0.00, 1.98]	2017
Fugii 2017	36	40	204	233	13.5%	1.28 [0.42, 3.86]	2017
**Total (95% CI)**		**501**		**560**	**100.0%**	**0.46 [0.25, 0.85]**	
Total events	319		463				

Heterogeneity: $Tau^2 = 0.42$; $Chi^2 = 18.15$, $df = 7$ ($P = 0.01$); $I^2 = 61\%$

Test for overall effect: $Z = 2.46$ ($P = 0.01$)

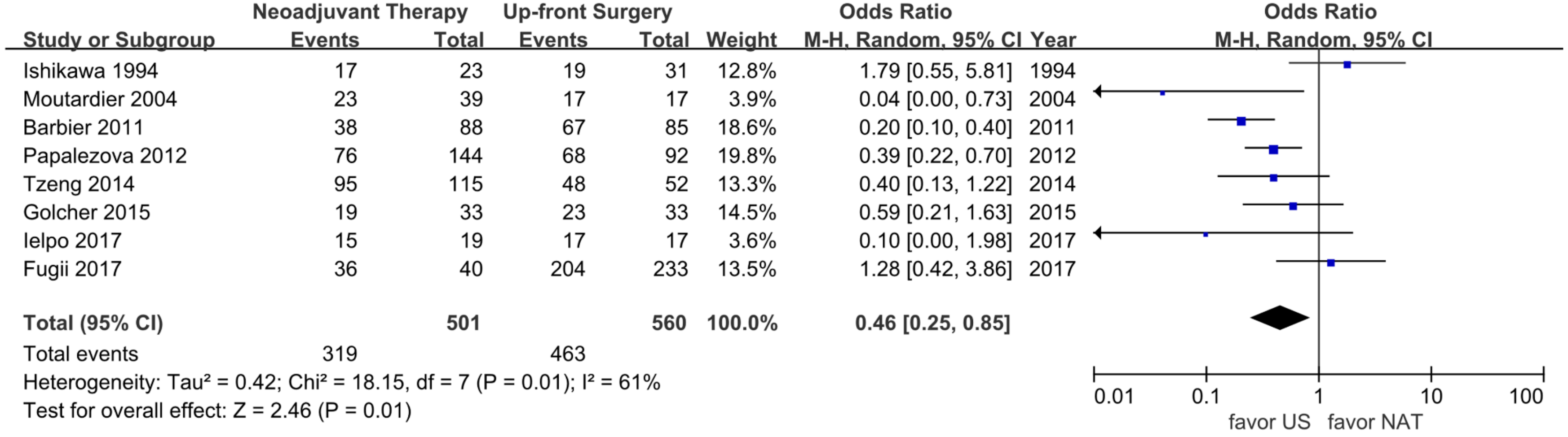

Figure S3. The rate of R0 resection after surgery was significantly higher in NAT group compared with US group (OR 1.53, 95% CI 1.35-1.73) and there was no statistically significant heterogeneity ($Chi^2 = 9.32$, $P = 0.230$, $I^2 = 25\%$).

	Neoadjuvant Therapy		Up-front Surgery			Odds Ratio	
Study or Subgroup	Events	Total	Events	Total	Weight	M-H, Fixed, 95% CI	Year
Moutardier 2004	21	23	11	17	0.3%	5.73 [0.99, 33.25]	2004
Barbier 2011	35	38	45	67	0.6%	5.70 [1.58, 20.61]	2011
Papalezova 2012	59	76	54	68	2.9%	0.90 [0.41, 2.00]	2012
Jiang 2013	95	112	96	120	3.3%	1.40 [0.71, 2.77]	2013
Tzeng 2014	85	95	39	48	1.3%	1.96 [0.74, 5.21]	2014
Golcher 2015	17	19	16	23	0.4%	3.72 [0.67, 20.63]	2015
Mokdad 2017	1670	2005	4688	6105	89.5%	1.51 [1.32, 1.72]	2017
Fugii 2017	34	40	163	204	1.9%	1.43 [0.56, 3.62]	2017
**Total (95% CI)**		**2408**		**6652**	**100.0%**	**1.53 [1.35, 1.73]**	
Total events	2016		5112				

Heterogeneity: Chi² = 9.32, df = 7 (P = 0.23); I² = 25%
Test for overall effect: Z = 6.77 (P < 0.00001)

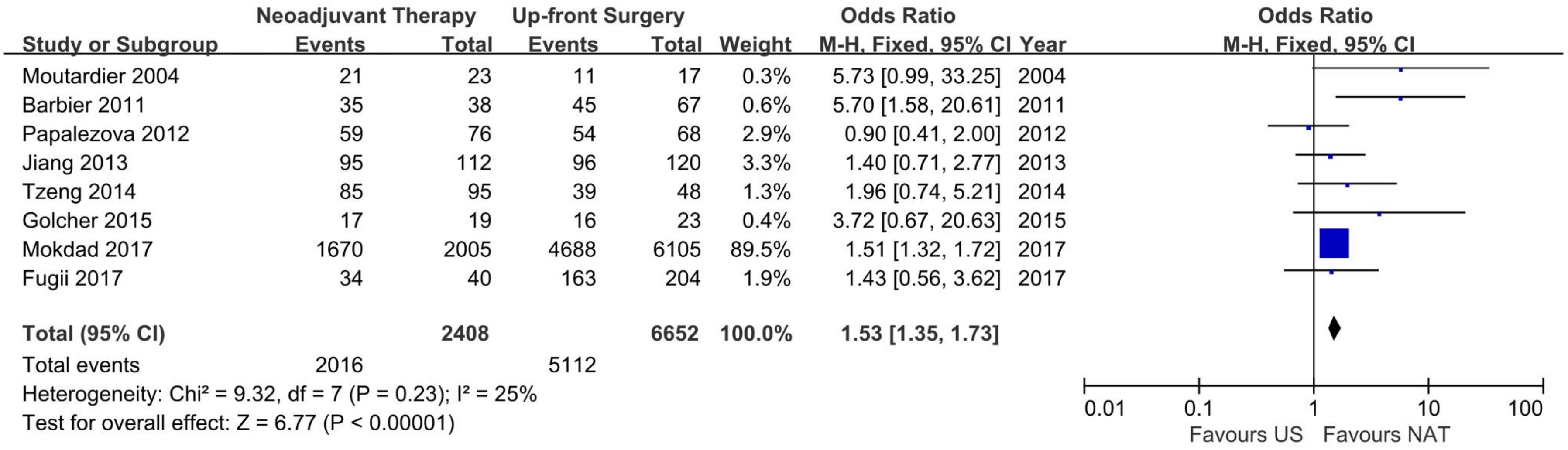

Figure S4 a. The rate of LN metastasis was significantly lower in NAT group compared with US group (OR 0.37, 95% CI 0.26-0.52, $P < 0.001$), although there was significant heterogeneity between studies ($Chi^2 = 21.04$, $P = 0.020$, $I^2 = 52\%$).

	Neoadjuvant Therapy		Up-front Surgery			Odds Ratio	
Study or Subgroup	Events	Total	Events	Total	Weight	M-H, Random, 95% CI	Year
Ishikawa 1994	5	17	4	18	4.2%	1.46 [0.32, 6.70]	1994
Moutardier 2004	3	23	11	17	4.0%	0.08 [0.02, 0.39]	2004
Vento 2007	7	15	5	14	4.4%	1.57 [0.35, 7.00]	2007
Barbier 2011	11	38	42	66	9.5%	0.23 [0.10, 0.55]	2011
Artinyan 2011	16	39	255	419	12.4%	0.45 [0.23, 0.87]	2011
Tajima 2012	10	13	12	21	4.1%	2.50 [0.53, 11.81]	2012
Papalezova 2012	19	76	42	68	11.6%	0.21 [0.10, 0.42]	2012
Tzeng 2014	49	95	39	48	9.9%	0.25 [0.11, 0.56]	2014
Golcher 2015	6	19	13	23	5.6%	0.36 [0.10, 1.27]	2015
Mokdad 2017	932	2005	4306	6105	22.6%	0.36 [0.33, 0.40]	2017
Fugii 2017	14	40	145	233	11.8%	0.33 [0.16, 0.66]	2017
**Total (95% CI)**		**2380**		**7032**	**100.0%**	**0.37 [0.26, 0.52]**	
Total events	1072		4874				

Heterogeneity: Tau² = 0.13; Chi² = 21.04, df = 10 (P = 0.02); I² = 52%
Test for overall effect: Z = 5.71 (P < 0.00001)

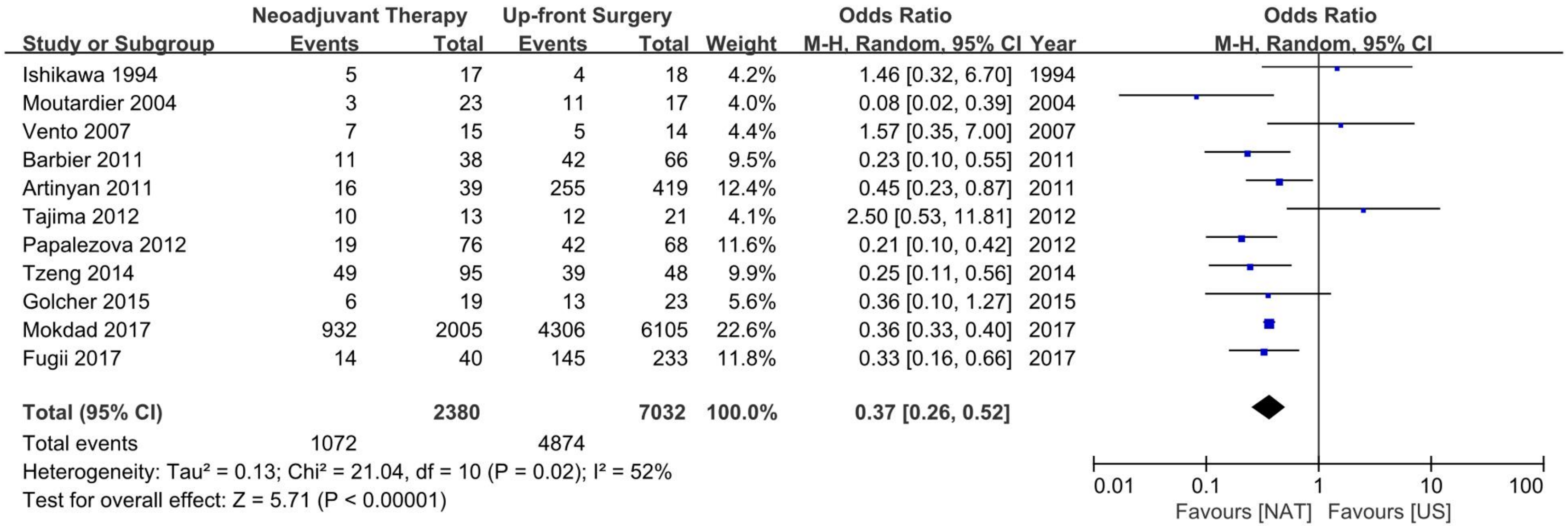

Figure S4 b. Sensitivity analysis showed that the favorable effect of NAT was still preserved (OR 0.32, 95% CI 0.24-0.43, *P* < 0.001) and the heterogeneity between studies statistically turned out to be insignificant ($Chi^2 = 11.78$, *P* = 0.160, $I^2 = 32\%$).

Study or Subgroup	Neoadjuvant Therapy Events	Neoadjuvant Therapy Total	Up-front Surgery Events	Up-front Surgery Total	Weight	Odds Ratio M-H, Random, 95% CI	Year
Ishikawa 1994	5	17	4	18	0.0%	1.46 [0.32, 6.70]	1994
Moutardier 2004	3	23	11	17	2.9%	0.08 [0.02, 0.39]	2004
Vento 2007	7	15	5	14	3.2%	1.57 [0.35, 7.00]	2007
Barbier 2011	11	38	42	66	8.2%	0.23 [0.10, 0.55]	2011
Artinyan 2011	16	39	255	419	12.1%	0.45 [0.23, 0.87]	2011
Tajima 2012	10	13	12	21	0.0%	2.50 [0.53, 11.81]	2012
Papalezova 2012	19	76	42	68	11.0%	0.21 [0.10, 0.42]	2012
Tzeng 2014	49	95	39	48	8.8%	0.25 [0.11, 0.56]	2014
Golcher 2015	6	19	13	23	4.3%	0.36 [0.10, 1.27]	2015
Mokdad 2017	932	2005	4306	6105	38.2%	0.36 [0.33, 0.40]	2017
Fugii 2017	14	40	145	233	11.3%	0.33 [0.16, 0.66]	2017
**Total (95% CI)**		**2350**		**6993**	**100.0%**	**0.32 [0.24, 0.43]**	
Total events	1057		4858				

Heterogeneity: $Tau^2 = 0.05$; $Chi^2 = 11.78$, df = 8 (P = 0.16); $I^2 = 32\%$

Test for overall effect: Z = 7.98 (P < 0.00001)

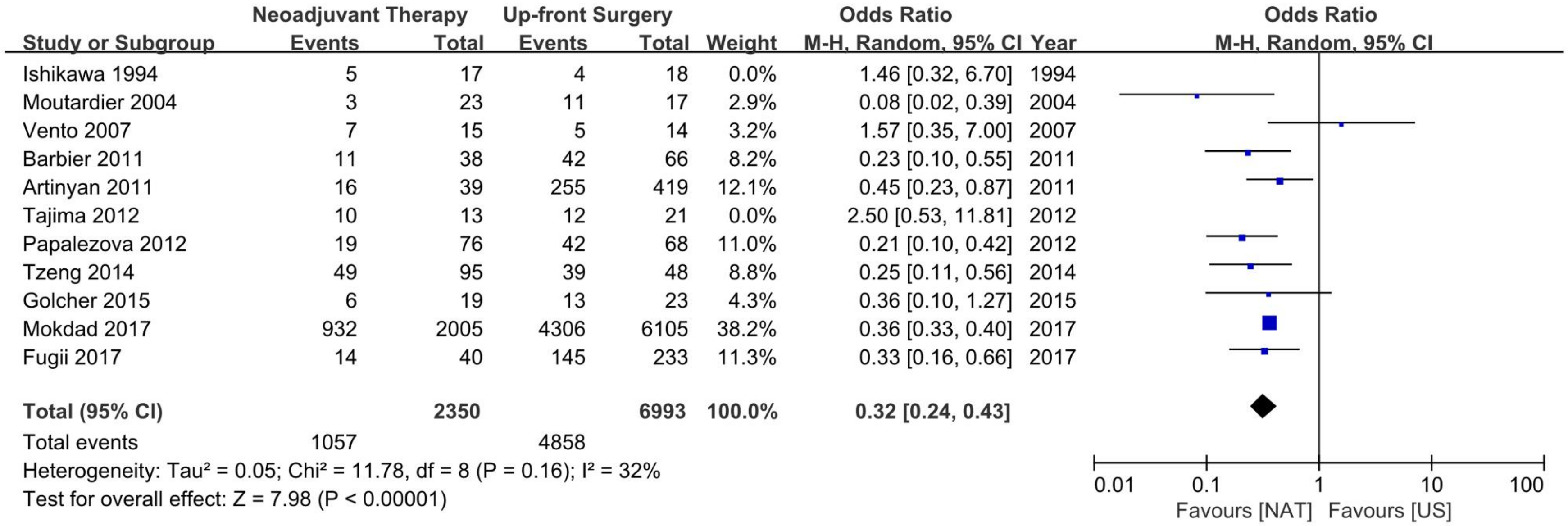

Figure S5. Funnel plots. (A) Overall survival, (B) lymph node metastasis (C) resection failure, (D) R0 resection.

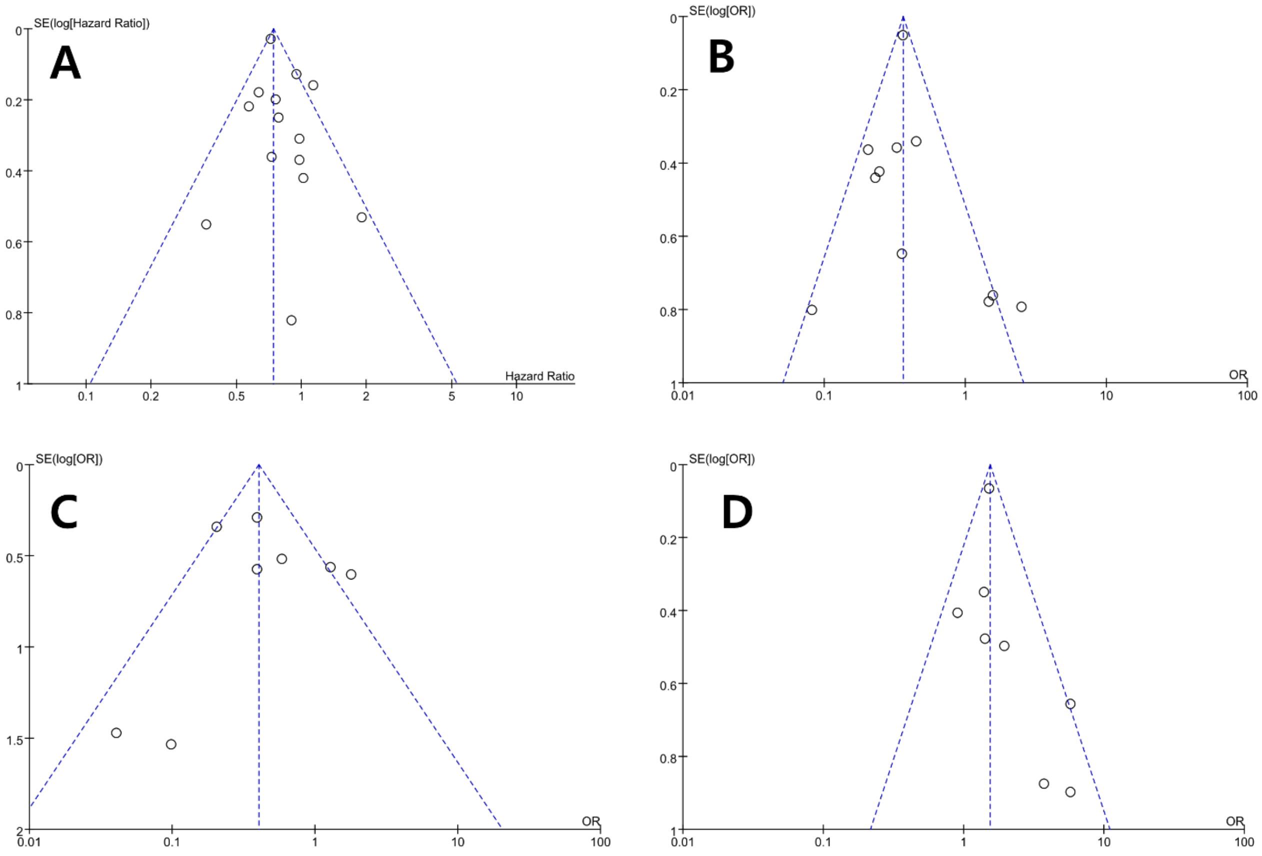

Supplement: Supplementary file 1 — Supplementary Information [file 41598_2019_52167_MOESM1_ESM.pdf]
